# Acute headache attributed to ischemic stroke: assessment of its characteristics and associated factors

**DOI:** 10.1055/s-0043-1763487

**Published:** 2023-04-14

**Authors:** Felipe Araújo Andrade de Oliveira, Mário Genuíno Dourado-Filho, Pedro Augusto Sampaio Rocha-Filho

**Affiliations:** 1 Universidade Federal de Pernambuco, Área Acadêmica de Neuropsiquiatria do Centro de Ciências Médicas, Recife PE, Brazil.; 2 Real Hospital Português de Beneficência de Pernambuco, Divisão de Neurologia, Recife PE, Brazil.

**Keywords:** Headache, Headache Disorders, Secondary, Vascular Headaches, Cerebral Infarction, Ischemic Stroke, Cefaleia, Transtornos Secundários da Cefaleia, Cefaleias Vasculares, Infarto Cerebral, AVC Isquêmico

## Abstract

**Background** It is estimated that headache attributed to ischemic stroke occurs in 7.4% to 34% of the cases. Despite its frequency, this headache has been little studied in terms of its risk factors and characteristics.

**Objective** To assess the frequency and clinical characteristics of headache attributed to ischemic stroke and the factors associated with its occurrence.

**Methods** The present was a cross-sectional study which included patients consecutively admitted within 72 hours of the onset of ischemic stroke. A semi-structured questionnaire was used. The patients underwent magnetic resonance imaging.

**Results** A total of 221 patients were included, 68.2% of whom were male, and the mean age was of 68.2 ±  13.8 years. The frequency of headache attributed to ischemic stroke was of 24.9% (95% confidence interval [95%CI]: 19.6–31.1%). The headache had a median duration of 21 hours and most frequently began at the same time as the focal deficit (45.3%), with a gradual onset (83%). It was of moderate intensity, pulsatile (45.3%), bilateral (54.6%), and presented a similar pattern to that of tension-type headache (53.6%). Headache attributed to stroke was significantly associated with previous tension-type headache, and previous migraine with and without aura (logistic regression).

**Conclusion** Headache attributed to stroke is common, with a pattern similar to that of tension-type headache, and it is associated with a history of tension-type and migraine headaches.

## Resumo

**Antecedentes** A cefaleia atribuída ao acidente vascular cerebral isquêmico (AVCi) tem uma frequência de 7,4% a 34% dos casos. Apesar de ser considerada frequente, esta cefaleia ainda é pouco estudada em termos de seus fatores de risco e características.

**Objetivo** Avaliar a frequência e as características clínicas da cefaleia atribuída ao AVCi e os fatores associados com a sua ocorrência.

**Métodos** Este foi um estudo transversal que incluiu pacientes admitidos consecutivamente com até 72 horas do início do AVCi. Foi utilizado um questionário semiestruturado. Os pacientes realizaram ressonância magnética.

**Resultados** Foram incluídos 221 pacientes, 68,2% dos quais eram do sexo masculino, e com idade média de 68,2 ±  13,8 anos. A frequência da cefaleia atribuída ao AVCi foi de 24,9% (intervalo de confiança de 95% [IC95%]: 19,6–31,1%). A duração mediana da cefaleia foi de 21 horas, e ela se iniciou com mais frequência ao mesmo tempo em que o déficit focal (45,3%), teve instalação gradual (83%), foi de moderada intensidade, pulsátil (45,3%), bilateral (54,6%) e teve um padrão semelhante ao da cefaleia de tipo tensional (53,6%). A cefaleia atribuída ao AVCi esteve significativamente associada à cefaleia de tipo tensional prévia, e à migrânea com e sem aura prévias (regressão logística).

**Conclusão** A cefaleia atribuída ao AVCi é frequente, tem padrão mais habitual semelhante ao da cefaleia de tipo tensional, e está associada aos antecedentes de cefaleia de tipo tensional e migrânea.

## INTRODUCTION

Headache attributed to ischemic stroke has been neglected and underestimated by both doctors and patients, 1 and, despite being frequent, it has been little studied in terms of its risk factors and characteristics. 2 The existing studies 2 have used various criteria to diagnose this headache, which generally differ from those of the International Classification of Headache Disorders. Furthermore, many of these studies have jointly assessed ischemic stroke and hemorrhagic stroke. 2

In order to diagnose headache attributed to stroke, the time between the onset of the headache and the development of the signs and symptoms of stroke should be short, and/or the headache must lead to a diagnosis of stroke, and/or an improvement in the headache should accompany a clinical improvement in the stroke. 3 This type of headache is estimated to occur in 7.4% to 34% of cases of stroke. 4 5 6 7 8 9 10 11 12 13 14 15 16 17 18 19

This headache is significantly more frequent in younger patients, 6 15 in those with migraine, 13 14 in cases of vertebrobasilar strokes, 9 11 14 16 and in cases of stroke with cortical rather than subcortical involvement. 4 11 Headache is believed to be more frequent in vertebrobasilar strokes due to a greater innervation of the meninges and vessels of the posterior fossa. 14 Moreover, there is a lower frequency of headache in strokes due to cerebrovascular disease of the small vessels (lacunar stroke) when compared to other etiologies. 4 10 14 16 18

In the few studies 11 15 20 that have assessed the characteristics of headache attributed to stroke, the reported frequency of pressure headache ranged from 15% to 66%, and that of pulsatile headache, from 8% to 80%. While pain of a mild to moderate intensity is most frequent, severe pain may occur in up to 26% of the patients. 11 21 Headache may be associated with nausea (28%), vomiting (6.5%), 11 20 and photophobia and phonophobia (24%), 22 and, on average, it lasts 29.5 ±  28 hours in cardioembolic strokes, 26.5 ±  18 hours in strokes due to large vessel atherosclerosis, and 19.5 ±  18 hours in lacunar strokes. 11

The objectives of the present study were to assess the frequency of headache attributed to stroke, its characteristics, and which patients or stroke characteristics are associated with the occurrence of this headache.

## METHODS

The present was a cross-sectional study.

### Patients

Patients with ischemic stroke were included in the study and were consecutively treated at the Real Hospital Português de Beneficência em Pernambuco, in the city of Recife, Northeastern Brazil. This is a private hospital with 850 beds, which is equipped with a neurological emergency and neurovascular unit. The patients were initially hospitalized in beds of the Neurological Intensive Care Unit (Neuro-ICU) and were subsequently transferred either to a private room or ward according to their clinical condition.

Data was collected from March 2017 to August 2020. Patients aged over 18 years and admitted within 72 hours of the onset of ictus were included. All patients underwent diffusion-weighted magnetic resonance imaging (DW-MRI). Ischemic stroke was diagnosed with the presence of a restricted diffusion sequence in the brain MRI within the context of a compatible clinical condition.

Patients who were unable to properly answer the questionnaire due to neurological deficit, such as those with aphasia and/or decreased level of consciousness, were excluded. Patients with a history of dementia were also excluded.

### Interview

The patients were assessed by a trained neurologist who conducted an interview using a semi-structured questionnaire containing questions on demographic data, the presence and characteristics of headaches throughout life, the presence and characteristics of headaches related to ischemic stroke, and the clinical condition related to stroke. All patients underwent clinical and neurological examinations.

The National Institutes of Health Stroke Scale (NIHSS) was applied upon admission to hospital; it measures neurological deficit, and the scores range from 0 to 42. The higher the score, the greater the neurological deficit. 23

For the etiological assessment of stroke, we used the classification system of the Trial of Org 10172 in Acute Stroke Treatment (TOAST). This classification system stratifies the etiology of cerebral infarctions into cardioembolic, atherosclerosis of large arteries, small vessel disease, another determined cause, and unknown cause. 24

Headaches were classified according to the diagnostic criteria of the third edition of the International Classification of Headache Disorders. Patients with migraine were those with previous headaches who met the diagnostic criteria for migraine with and without aura and probable migraine, and patients with tension-type headache were those with previous headaches who met the diagnostic criteria for tension-type headache and probable tension-type headache. 3

A diagnosis of headache attributed to stroke was determined by the existence of a headache with a diagnosis of stroke. The causal relationship was determined by the time between the onset of headache and the development of signs and symptoms of stroke, or when the headache led to a diagnosis of stroke. 3 In the present study, for the purposes of diagnosis, we considered headaches that began between the 24 hours preceding the focal deficits of stroke and up to 7 days after the stroke.

The International Classification of Headache Disorders ^3^ does not determine the exact time to diagnose, it only indicates that there must be a very close temporal relationship between the onset of headache and the diagnosis of ischemic stroke. For other secondary types, such as headache attributed to traumatic injury to the head, those attributed to craniotomy, and postendarterectomy headache, the International Classification of Headache Disorders has used a period of up to 7 days between events, which was the one we decided to use. 25

### Magnetic resonance

All patients underwent brain DW-MRI, which was standardized according to the service protocol, using Siemens 1.5 T scanners (Siemens Healthineers AG, Erlangen, Germany). The following parameters were used in the diffusion sequence: TR 3600 ms, TE 92 ms, and voxel 1.9 × 1.9 × 5 mm.

The ischemic volume and the area affected by ischemia were assessed by a radiologist from the hospital's service. With the use of a specific software (Horus, Horus Software GmbH, Ettlingen, Baden-Wurttemberg, Germany), the radiologist manually delimited the ischemic area in each slice, and the software subsequently calculated the total volume. The MRI diffusion sequence was used to determine this piece of information. The radiologist had no access to information regarding the patient's diagnoses or headaches.

In patients with small infarctions, which were only visualized in one axial slice in the diffusion sequence, a three-dimensional analysis of the volume was impossible, and these patients were excluded only from this specific analysis of the volume.

### Statistical analysis

The statistical analyzes were performed using the STATA (StataCorp, College Station TX, United States) software, version 14. The statistical association of the nominal variables was obtained using the Pearson Chi-squared test.

The Kolmogorov-Smirnov test was used to assess normality. The statistical association of the continuous variables with normal distribution was assessed though the Student *t* -test. The statistical association of the continuous variables with no normal distribution was assessed through the Mann–Whitney test.

The patients were divided into two groups: those with headache attributed to stroke and those without. Both were compared in terms of their demographic characteristics and clinico-radiological characteristics.

Headache attributed to stroke was considered as having a “migraine pattern” if its characteristics met criteria C and D for migraine without aura, and a “tension-type headache pattern” if its characteristics met criteria C and D for episodic tension-type headache. 3

For the multivariate analysis, a logistic regression model was applied to estimate the factors associated with headache. Stepwise modeling was used, and variables in the bivariate analysis entered the model following the order of statistical significance (forward selection). The criterion to entering the variables in the model was values of *p* < 0.20, and, for the output variables of the model, *p* > 0.15. Statistical significance was set as values of *p* < 0.05.

### Ethical considerations

All patients signed the informed consent form. The present study was approved by the Research Ethics Committee at the Universidade Federal de Pernambuco (under CAAE: 63479916.3.0000.5208; approval number: 2.394.830), in accordance with the ethical principles of the Declaration of Helsinki.

## RESULTS

**Figure 1:**
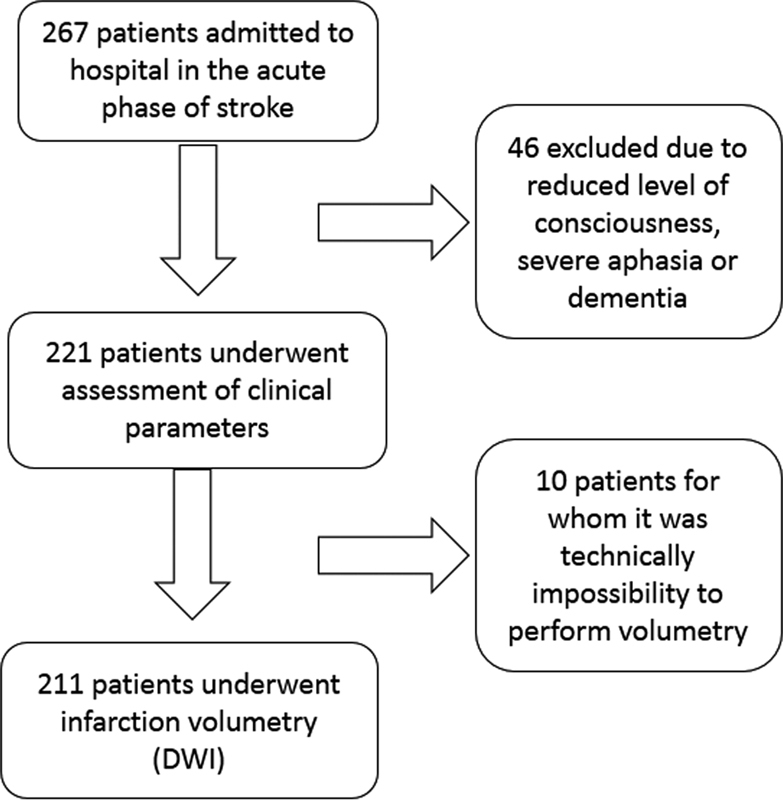
Flowchart of patients assessed for ischemic stroke.

Figure 1 represents the flowchart of the study.

The demographic and clinical characteristics of the patients are presented in Table 1 . Most of the patients were older (mean age: 68.2 years), male (59,3%), and white (65.6%). The main causes of stroke were small vessel disease (26.2%) and cardiac embolism (28.5%), and there was a higher frequency of anterior circulation stroke (63.3%). There were two cases of ischemic stroke caused by an arterial dissection.

**Table 1:** Clinical characteristics of 221 stroke patients

Characteristics		Statistics
Time since admission (hours): median (P _25_ –P _75_ )		6 (2.5–22)
NIHSS score on admission: median (P _25_ –P _75_ )		2 (1–5)
Etiology: n (%)	Large vessel atherosclerosis	24 (10.9%)
	Small vessel disease	58 (26.2%)
	Cardiac embolism	63 (28.5%)
	Other determined etiologies	16 (7.2%)
	Undetermined	60 (27.2%)
Hyperacute phase treatment: n (%)	None	194 (87.8%)
	Chemical thrombolysis	10 (4.5%)
	Mechanical thrombectomy	11 (5.0%)
	Chemical thrombolysis in association with mechanical thrombectomy	6 (2.7%)
	Ischemic location: cortical involvement	117 (52.9%)
Circulation affected by ischemia: n (%)	Anterior	140 (63.3%)
	Posterior	66 (29.9%)
	Both	15 (6.8%)
Side affected by ischemia: n (%)	Left	100 (45.2%)
	Right	108 (48.9%)
	Bilateral	13 (5.9%)
Volumetry (cm ^3^ ): median (P _25_ –P _75_ )		1.58 (0.34–8.4)
History of systemic arterial hypertension: n (%)		165 (74.7%)
History of coronary artery disease: n (%)		32 (14.5%)

Abbreviation: NIHSS, National Institutes of Health Stroke Scale.

Headache attributed to stroke occurred in 55 patients (24.9%; 95% confidence interval [95%CI]: 19.6–31.1%). In one of these patients, the headache led to a diagnosis of stroke. Table 2 presents the clinical characteristics of this headache. Two patients were unable to remember the characteristics of their headaches and were not included in this analysis. Headache was more often gradual (83%), with concomitant onset of the cerebrovascular event (45.3%), of moderate intensity (median: 6), bilateral (54.6%), and with a pattern similar to that of tension-type headache (63.6%). In most patients, the headache either began with a focal deficit or during the 24 hours before or after the event.

**Table 2:** Clinical characteristics of headache attributed to stroke (n = 53 patients)

Characteristics		Data
Temporal relationship with the event: n (%)	Before	10 (18.9%)
	Concomitantly	24 (45.3%)
	After	19 (35.8%)
Onset: n (%)	Sudden	9 (17.0%)
	Gradual	44 (83.0%)
Associated symptoms: n (%)	Photophobia	21 (38.9%)
	Phonophobia	16 (29.6%)
	Nausea	16 (29.6%)
	Vomiting	6 (11.1%)
	Becomes worse with effort	9 (16.7%)
	Autonomic symptoms	11 (20%)
	Conjunctival hyperemia	6 (11.3%)
	Tearing	9 (17%)
	Nasal congestion	3 (5.7%)
	Rhinorrhea	1 (1.9%)
	Sweating	1 (1.9%)
	Duration (hours): median (P _25_ –P _75_ )	21 (2–60)
	Intensity: Median (P _25_ –P _75_ )	6 (4–7)
Laterality: n (%)	Bilateral	30 (54.6%)
	Unilateral	25 (45.4%)
	Right	19 (76.0%)
	Left	6 (24.0%)
Location: n (%)	Frontal	29 (52.7%)
	Temporal	16 (29.1%)
	Parietal	12 (21.8%)
	Occipital	18 (32.7%)
Characteristics of the headache: n (%)	Pulsatile	24 (45.3%)
	Pressure	20 (37.7%)
	Stabbing	2 (3.8%)
	Others	7 (13.2%)
Pattern of the headache: n (%)	Migraine	19 (34.6%)
	Tension	35 (63.6%)
	Not classifiable	1 (1.8%)

History of migraine was present in 22 patients, who reported headache during the acute phase of stroke. Of these patients, 12 presented headache with a “migraine pattern”. Previous tension headaches and headache during the acute phase of the stroke was reported by 15 patients, 9 of whom presented a headache with “tension-type pattern”.

Right-sided stroke was present in 30 patients, 15 of whom presented with unilateral headache on the right side, and 15 presented with bilateral headache. A total of 19 patients presented with stroke on the left side and, of these, 4 reported unilateral headache on the left side, 4, unilateral headache on the right side, and 11 presented with bilateral headaches. Of the 6 patients with bilateral stroke, 2 reported unilateral headaches on the left side and 4 patients reported bilateral headaches.

Table 3 presents the characteristics of the patients and the stroke that are associated with headache attributed to stroke. After controlling for confounding variables, there was a significant association between the occurrence of headache attributed to stroke and history of migraine with and without aura and tension-type headache.

**Table 3:** Association of characteristics with the occurrence of headache attributed to stroke

Characteristics		With headache (n = 55): n (%)	Without headache (n = 166): n (%)	OR (95%CI)	*p* -value	OR _adjusted_ (95%CI)	*p* -value _adjusted_
Sex	Male	28 (50.9%)	103 (62.0%)	Reference	−		
	Female	27 (49.1%)	63 (38.0%)	1.58 (0.85–2.91)	0.147		
Age (years): mean ± standard deviation		66.0 ± 14.7	68.9 ± 13.4	0.98 (0.96–1.01)	0.174		
Skin color	White	33 (60.0%)	112 (67.5%)	Reference	−		
	Non-white	22 (40.0%)	54 (32.5%)	1.38 (0.74–2.59)	0.313		
NIHSS score on admission		2 (1–4)	2 (1–5)	0.93 (0.85–1.01)	0.101		
Cortical involvement		37 (67.3%)	80 (48.2%)	2.21 (1.16–4.19)	0.015	1.81 (0.86–3.85)	0.119
Circulation affected by ischemia	Anterior	30 (54.6%)	110 (66.3%)	Reference	−	Reference	−
	Posterior	18 (32.7%)	48 (28.9%)	1.37 (0.70–2.70)	0.356	1.66 (0.75–3.67)	0.211
	Both	7 (12.7%)	8 (4.8%)	3.21 (1.08–9.56)	0.036	3.16 (0.94–10.7)	0.063
Volumetry (cm ^3^ )			1.4 (0.3–7.5)	1.01 (0.99–1.02)	0.541		
Etiology of the stroke	Cardiac embolism	14 (25.5%)	49 (29.5%)	Reference	−		
	Large vessel atherosclerosis	10 (18.2%)	14 (8.4%)	2.5 (0.91–6.83)	0.074		
	Small vessel diseases	7 (12.7%)	51 (30.7%)	0.48 (0.18–1.29)	0.146		
	Other etiologies determined	6 (10.9%)	10 (6.1%)	2.1 (0.65–6.79)	0.215		
	Undetermined	18 (32.7%)	42 (25.3%)	1.5 (0.67–3.37)	0.327		
History of systemic arterial hypertension		35 (63.6%)	130 (78.3%)	0.48 (0.25–0.94)	0.032	0.46 (0.21–1.01)	0.056
History of coronary arterial disease		6 (10.9%)	26 (15.7%)	0.66 (0.26–1.70)	0.388		
History of migraine		22 (40.0%)	37 (22.3%)	2.32 (1.21–4.46)	0.011		
With or without aura	No history of migraine	33 (60.0%)	129 (77.7%)	Reference	−	Reference	−
	Migraine with aura	12 (21.8%)	14 (8.4%)	3.35 (1.42–7.92)	0.006	5.07 (1.92–13.4)	0.001
	Migraine without aura	10 (18.2%)	23 (13.9%)	1.70 (0.74–3.92)	0.213	2.73 (1.06–7.08)	0.038
History of tension-type headache	No tension-type headache	40 (72.8%)	157 (94.6%)	Reference	−	Reference	−
	Tension-type headache	15 (27.2%)	9 (5.4%)	6.54 (2.67–16.0)	< 0.001	8.88 (3.28–24.0)	< 0.001

Abbreviations: 95%CI, 95% confidence interval; NIHSS, National Institutes of Health Stroke Scale; OR, odds ratio.

## DISCUSSION

In the present study, we found a frequency of 24.9% of headache attributed to ischemic stroke, which is in line with reports in the literature (range: 7.4% to 34%). 4 5 6 7 8 9 10 11 12 13 14 15 16 17 18 19

With regard to its characteristics, the headache most often began at the same time as the focal deficit, and the onset was predominantly gradual. Previous studies have reported a higher frequency of headache that began before the stroke (range: 26% to 60% of the cases), 11 19 21 26 with a similarity between sudden and gradual onset. 5 21 However, these studies included both ischemic and hemorrhagic strokes. A higher frequency of sentinel headaches and the sudden onset of pain amongst patients with hemorrhagic stroke may have contributed to this difference.

We observed that headaches were predominantly of a moderate intensity, with both bilateral and frontal pain, and the most frequent pattern was similar to that of tension-type headache, which has also been observed in other studies. 11 15 20 21

The median duration of the headache was of 21 hours. Only 1 study 11 assessed the duration of the headache, and the authors reported a duration very similar to that of the present study (mean: 25 ±  28 hours).

Photophobia was observed in 38.9% of the patients, and phonophobia, in 29.6%. Another study 20 grouped these conditions together and reported a frequency of 24%. Nausea was observed in 29.6% and vomiting, in 11.1% of our patients with headache, frequencies similar to those reported in the literature. 11 20 22 Autonomic symptoms were observed in 20% of our patients with headache. Seifert et al. 20 reported a frequency of 24%. These symptoms associated with headache may be related to the location of the ischemia. 20

No association was identified between the presence of headache attributed to stroke and gender, which has been a controversial point in the literature. 4 6 14 15 Although a younger age was not statistically significant in the multivariate analysis ( *p* = 0.086), it remained in the model for the presence of headache attributed to stroke. Two other studies 6 15 reported a higher frequency of headache attributed to stroke among younger patients. We cannot rule out the possibility that the statistical power of the present study was insufficient to detect this difference in relation to age.

Neither were there differences in relation to an association between neurological deficit (NIHSS) and the presence of headache attributed to stroke. Another study 20 with a design similar to that of ours did not report associations either. In two other studies, 10 16 this headache was associated with lower NIHSS scores. However, these were retrospective studies, which therefore makes comparison difficult. Studies 4 10 14 16 18 have demonstrated that patients with lacunar stroke, who usually present lower neurological deficit, also present a lower frequency of headache attributed to stroke.

To the best of our knowledge, the present is the first study to assess whether there is an association between headache and lesion volume. We have observed no association. Seifert et al. 20 reported an association between the presence of headache and infarctions located in areas related to processing pain, such as the insula and the somatosensory cortex. Our finding reinforces the hypothesis that the strategic location of the stroke is more critical to generate headache than its volume.

The presence of cortical infarction ( *p* = 0.119) and stroke with concomitant impairment of the anterior and posterior circulations ( *p* = 0.065) remained in the multivariate analysis model and were important to explain the distribution of headache attributed to stroke. An association between headache and cortical infarctions has already been described. 4 11 20 There is biological plausibility for this, since cortical lesions may trigger widespread cortical depression, even in individuals without migraine. 27 It is possible that cortical strokes from different circulatory systems further increase the likelihood of having widespread cortical depression.

Other studies 9 11 13 14 16 have reported a higher frequency of headache in posterior circulation stroke when compared to anterior circulation stroke. We did not observe this association. Most of these studies did not use MRI; 11 13 14 16 therefore, they may not have been able to detect small infarctions, in addition to being more subject to topographic classification errors. One study, 20 similar to ours, that used brain MRI, did not find a higher frequency of headache in areas irrigated by the posterior system either.

We have also observed an association between the diagnoses of migraine with and without aura and the presence of headache attributed to stroke, which was stronger regarding migraine with aura. This finding is corroborated by other studies, 14 15 although they did not conduct a separate analysis of migraine with and without aura. Other studies 25 28 have also linked the presence of previous primary headaches with the development of secondary headaches. The presence of migraine predisposes to the phenomenon of widespread cortical depression in individuals with stroke, with consequent activation of the trigeminovascular system. 29 This may trigger pain. 30

We have observed a significant association of headache attributed to stroke and a diagnosis of previous episodic tension-type headache. Another study 20 did not report such an association; however, this study assessed a smaller number of patients. Although the pathophysiology of tension-type headache has not yet been well established, it is believed that these patients have central sensitization to pain and hypersensitivity to stimuli, especially those with chronic tension-type headache. 31 It is possible that this hypersensitization increases the chance of triggering headache after the ischemic insult.

Although the presence of migraine and tension-type headache appeared as risk factors for headache attributed to stroke, there was a low correlation between the characteristics of the previous primary headache and the pattern of headache attributed to stroke. This reveals that the patients did not present an attack of their primary headache during the stroke and corroborates the existence of the headache attributed to stroke as an autonomous clinical entity. There was also a low correlation between the side of the ischemia and the side of the headache, which has also been reported by another study. 20

No association was observed between the headache and the etiology of the stroke. Four previous studies 4 10 14 16 have reported a lower frequency of headache in patients with a lacunar stroke. However, two of these studies 4 10 did not perform multivariate analyses to control for confounding variables. One study 16 only adjusted the analysis only for age, sex, and the presence of arterial hypertension, and another study 14 included symptoms associated with stroke and headache in the analysis, thereby making comparisons with our results difficult.

The present study has some limitations. It was a single-center study with a convenience sample. Our population had higher socioeconomic status than that of the average of the Brazilian population. The strokes included were mostly of small volume and with minor neurological deficits. Patients with more severe ischemic strokes who could not respond to the questionnaire were excluded. This decreases the generalizability of the study. The sample size was not calculated and the number of patients in the statistical analysis could be considered low. Thus, we cannot rule out the possibility that we have been unable to detect small differences. The diagnosis of migraine and tension-type headache was made close to the occurrence of the stroke. Thus, we cannot rule out the possibility that we have not identified individuals with a low frequency of these headaches.

On the other hand, the present study has several strengths. The patients were assessed by a neurologist, and all of them underwent clinical, neurological and magnetic resonance imaging examinations. The radiologist was unaware of the patients' clinical data. The International Classification of Headache Disorders was used to diagnose the headaches. These measures were taken in an attempt to decrease the classification errors of the study and increase its internal validity.

In conclusion, headache attributed to stroke is a frequent manifestation, and it generally has a gradual onset. It is bilateral, pulsatile, of a moderate intensity, and begins concomitantly to the focal deficit. Migraine and tension-type headache are risk factors for headache attributed to ischemic stroke and often demonstrate a different pattern from the previous primary headache in these patients.
